# A comparison between tau and amyloid-β cerebrospinal fluid biomarkers in chronic traumatic encephalopathy and Alzheimer disease

**DOI:** 10.1186/s13195-022-00976-y

**Published:** 2022-02-09

**Authors:** Katherine W. Turk, Alexandra Geada, Victor E. Alvarez, Weiming Xia, Jonathan D. Cherry, Raymond Nicks, Gaoyuan Meng, Sarah Daley, Yorghos Tripodis, Bertrand R. Huber, Andrew E. Budson, Brigid Dwyer, Neil W. Kowall, Robert C. Cantu, Lee E. Goldstein, Douglas I. Katz, Robert A. Stern, Michael L. Alosco, Jesse Mez, Ann C. McKee, Thor D. Stein

**Affiliations:** 1 Boston University Alzheimer’s Disease Research and CTE Center, Boston University School of Medicine, Boston, MA 02118 USA; 2 VA Boston Healthcare System, 150 S. Huntington Avenue, Boston, MA 02130 USA; 3 Department of Neurology, Boston University School of Medicine, Boston, MA 20118 USA; 4 Boston University School of Medicine, Boston, MA 02118 USA; 5 VA Bedford Healthcare System, Bedford, MA 01730 USA; 6 Department of Biostatistics, Boston University School of Public Health, Boston, MA 20118 USA; 7 Braintree Rehabilitation Hospital, Braintree, MA 02118 USA; 8 Department of Anatomy and Neurobiology, Boston University School of Medicine, Boston, MA 20119 USA; 9 Concussion Legacy Foundation, Boston, MA 02115 USA; 10 Department of Neurosurgery, Boston University School of Medicine, Boston, MA 02118 USA; 11 Department of Neurosurgery, Emerson Hospital, Concord, MA 01742 USA; 12 Departments of Psychiatry, Ophthalmology, Boston University School of Medicine, Boston, USA; 13 Departments of Biomedical, Electrical & Computer Engineering, Boston University College of Engineering, Boston, USA; 14 Department of Pathology and Laboratory Medicine, Boston University School of Medicine, Boston, MA 02118 USA

**Keywords:** Cerebrospinal fluid, Biomarkers, Chronic traumatic encephalopathy, Alzheimer’s disease, Amyloid beta, Tau

## Abstract

**Background:**

Cerebrospinal fluid (CSF) tau and beta-amyloid levels in chronic traumatic encephalopathy (CTE), a disease which can be clinically indistinguishable from Alzheimer’s disease (AD), are largely unknown. We examined postmortem CSF analytes among participants with autopsy confirmed CTE and AD.

**Methods:**

In this cross-sectional study 192 participants from the Boston University AD Research Center, VA-BU-CLF Center, and Framingham Heart Study (FHS) had post-mortem CSF collected at autopsy. Participants were divided into pathological groups based on AD and CTE criteria, with 61 CTE participants (18 low, 43 high stage), 79 AD participants (23 low, 56 intermediate to high), 11 participants with CTE combined with AD, and 41 participants lacking both CTE and AD neuropathology. The Meso Scale Discovery immunoassay system was utilized to measure amyloid-beta (Aβ_1-40,_ Aβ_1-42_)_,_ total tau (t-tau), and phosphorylated tau (p-tau_181_ and p-tau_231_). CSF analytes were then compared across the pathological groups: no CTE/no AD (control), Low CTE, Low AD, High CTE, Intermediate/High AD, and AD+CTE.

**Results:**

Among the Low disease state groups, the Low CTE group had significantly higher levels of p-tau_231_ versus the control group and compared to the Low AD group. The Low CTE group was also found to have significantly lower levels of Aβ_1-42_ compared to the control group. The high CTE group had higher levels of p-tau_231_ and lower levels of Aβ_1-42_ compared to Intermediate/High AD group.

**Conclusions:**

Importantly, p-tau_231_ and Aβ_1-42_ were predictors of diagnosis of CTE vs. control and CTE vs. AD. Increased CSF p-tau_231_ is a promising potentially sensitive biomarker of CTE, and CSF Aβ_1-42_ needs further investigation in CTE.

**Supplementary Information:**

The online version contains supplementary material available at 10.1186/s13195-022-00976-y.

## Background

Chronic traumatic encephalopathy (CTE) and Alzheimer disease (AD) are neurodegenerative conditions causing memory loss that can only be definitively diagnosed by pathologic examination. CTE is neuropathologically distinct from AD and characterized by hyperphosphorylated tau (p-tau) deposition in neurons, astrocytes, and cell processes around the small blood vessels and at the depths of cortical sulci that often involves the superficial cortical layers II and III [1–3]. AD neuropathology consists of amyloid-β plaques and tau neurofibrillary tangles in a distinct pattern beginning in the brainstem and entorhinal cortex in the early stages and progressing to the medial temporal lobe and finally the neocortex [4]. Analysis of differential profiles of tau-epitope phosphorylation profiles between CTE and AD is not yet fully understood though elevated levels of p-tau_181_ and p-tau_231_ have been reported in both AD and CTE [5, 6]. Total tau (t-tau), phosphorylated tau (p-tau), and beta-amyloid (Aβ) measurements in cerebrospinal fluid (CSF) have proven to be reliable biomarkers of AD and reflect changes in brain pathology that precede cognitive decline ante-mortem [7–10]. Biomarkers with the ability to detect and distinguish CTE from AD would be of great value if they show a high degree of correlation with CTE neuropathology.

Given the relatively superficial location of p-tau in the cortex of patients with CTE compared to AD and the lack of notable involvement of amyloid β in CTE, we hypothesized that CTE participants may have increased t-tau, p-tau_181_, and p-tau_231_ compared to cases without either CTE or AD (control), as well as relatively higher p-tau_181_, p-tau_231_, and Aβ_42_ concentrations compared to AD cases. Prior studies of Aβ deposition in CTE [11] led us to hypothesize that CSF Aβ_1-40_ would be decreased in cases with co-occurring CTE and AD compared to non-AD/non-CTE cases, representing a potential interaction between AD and CTE. To test these hypotheses, we measured CSF levels of Aβ_1-40_, Aβ_42_, t-tau, p-tau_181_, and p-tau_231_ among autopsy-confirmed participants including 61 with CTE, 79 with AD, 11 with concurrent CTE and AD, and 41 control participants lacking both CTE and AD pathology. Low and high stage CTE involve different brain regions and are associated with different clinical symptoms such that high stage disease shows significant medial temporal lobe pathology and is associated with increased frequency of cognitive impairment and dementia [12]. In contrast, intermediate to high degrees of AD pathology are characterized by greater pathological involvement of the neocortex and are also associated with cognitive impairment and dementia [13]. Therefore, because the underlying neuropathology and clinical syndrome is markedly different in low and high stage disease [12], we compared CTE to AD CSF analytes separately in each disease stage.

## Methods

### Participants

One hundred ninety-two participants with post-mortem CSF available were enrolled from three study groups. 100 donated their brains to the Veteran’s Affairs-Boston University-Concussion Legacy Foundation Brain Bank (VA-BU-CLF) as part of the Understanding Neurologic Injury and Traumatic Encephalopathy (UNITE) study, 38 were donated to the Framingham Heart Study (FHS), and 54 were donated to Boston University’s Alzheimer’s Disease Research Center (ADRC) as part of the Health Outreach Program for the Elderly study. The UNITE group consisted of participants with a history of exposure to contact sports such as football, ice hockey, boxing, soccer, rugby, and martial arts at either the professional or amateur level [14]. For most brain donations, the next of kin contacted the brain bank to donate tissue at or near the time of death. The participants from Boston University’s Alzheimer’s Disease Research Center (BU ADRC) with and without cognitive impairment underwent annual cognitive evaluations using the National Alzheimer’s Disease Coordinating Center (NACC) Uniform Data Set (UDS) protocol [1]. The third cohort consisted of participants from the Framingham Heart Study (FHS), a longitudinal, community-based study. Consents for brain donation and research participation were provided by donor next of kin.

### Head injury exposure assessment

For UNITE study participants, retrospective clinical evaluations were performed using semi-structured post-mortem interviews, through online surveys, and review of medical records as described previously [14]. Information was obtained regarding repetitive head impact (RHI) exposure, traumatic brain injury (TBI) exposure, military history, athletic history, and clinical symptoms prior to death. In addition, medical records were examined to provide a determination of clinical symptoms and course. For the FHS participants, an athletic history assessment identical to UNITE was performed with the donor’s next of kin [15]. Athletic history was not available for BU ADRC participants. All interviews were conducted independently and blinded to the results of neuropathological examination.

### Pathological criteria

All brains were neuropathologically evaluated for changes consistent with CTE, AD, and other neurodegenerative disorders using previously described selection criteria and protocols. Specifically, participants were separated into pathologic groups: CTE, AD, both (CTE+AD), or neither no CTE/no AD (control). Pathologic diagnosis of CTE was based on consensus criteria [1] and CTE staging I–IV was determined using previously published staging criteria [12, 16]. Participants were stratified using NIA-Reagan criteria to high, intermediate, or low probability of dementia caused by AD, based on Braak Score and CERAD score.

There is growing recognition that with increasing age co-occurring neurodegenerative pathologies become more common [17, 18]; therefore, comorbid neurodegenerative pathologies were not excluded from any of the groups, including the control group. Other neurodegenerative diseases were diagnosed using well-established criteria for Lewy body disease (LBD) [19] and frontotemporal lobar degeneration (FTLD) [20, 21] (Table 1). Participants with Amyotrophic Lateral Sclerosis/motor neuron disease (ALS/MND) pathology were also not excluded and included two participants in the no CTE/no AD (control) group and one participant in the high CTE group. The inclusion of a wider group of participants with concurrent neurodegenerative pathologies increases the generalizability of the current findings as patients presenting for clinical evaluation of cognitive complaints often have multiplane neurodegenerative diagnoses and underlying pathologies.

**Table 1:** Demographic and exposure characteristics of participant groups

	No CTE/No AD	Low CTE	High CTE	Low AD	Int/High AD	CTE+AD	***p***
**Sample size (*n*)**	41	18	43	23	56	11	
**Age at death (S.E.M.)**	74.1 (3.0)^d^	64.6 (4.6)^f,d,e^	74.8 (1.3)^d^	86.7 (1.1)^a,b,c^	81.1 (1.3)^b^	79.6 (2.8)^b^	<.001
**Age range (max-min)**	101–17	89–25	90–53	95–72	98–54	70–100	
**Cohort**							
FHS	13	1	0	10	13	1	
UNITE	17	17	43	1	12	10	
HOPE	11	0	0	12	31	0	
Sex m/f (%male)	33/8 (80.5%)^c,e^	18/0 (100%)^e^	43/0 (100%)^a,d,e^	14/9 (60.9%)^c^	27/29 (48.2%) ^a,b,c^	10/1 (90.9%)	<.001
**CTE stage**							
Stage I	0	6 (33.3%)	0	0	0	1 (9.1%)	
Stage II	0	12 (66.7%)	0	0	0	1 (9.1%)	
Stage III	0	0	18 (41.9%)	0	0	0	
Stage IV	0	0	25 (58.1%)	0	0	9 (81.8%)	
**Braak Score**							
0	15 (36.6%)	7 (38.9%)	1 (2.3%)	0	0	0	
I-II	12 (29.3%)	5 (27.8%)	4 (9.3%)	6 (26.1%)	0	0	
III-IV	14 (34.1%)	5 (27.8%)	31 (72.1%)	17 (73.9%)	7 (12.5%)	1 (9.1%)	
V-VI	0	1 (5.6%)	7 (16.3%)	0	49 (87.5%)	10 (90.9%)	
**CERAD Score**							
0	41 (100%)	14 (77.8%)	21 (48.8%)	0	0	0	
1	0	4 (22.2%)	22 (51.2%)	22 (95.7%)	8 (14.3%)	1 (9.1%)	
2	0	0	0	1 (4.3%)	26 (46.4%)	6 (54.5%)	
3	0	0	0	0	22 (39.3%)	4 (36.4%)	
**FTLD pathology**	10 (27.7%)^d,e^	5 (27.8%)^d,e^	6 (14.6%)	0 (0%)^a,b^	3 (5.4%)^a,b^	2 (18.2%)	<.05
**Tau**	9 (25%)	5 (27.8%)	6 (14.6%)	0	2 (4.3%)	2 (18.2%)	
**TDP 43**	1 (2.7%)	5 (27.8%)	4 (9.8%)	0	2 (4.3%)	1 (9%)	
***n*** **total**	36	18	41	15	46	11	
**LBD pathology**							0.31
Brainstem	2 (4.4%)	1 (5.6%)	6 (14%)	2 (13.3%)	2 (4.34%)	0 (0%)	
Limbic/neocortical	4 (9.8%)	2 (11.1%)	10 (23.3%)	1 (6.6%)	8 (17.4%)	4 (36.4%)	
***n***	36	18	43	15	46	11	
**Contact sports play**	Yes 12 (29.2%)	Yes 17 (94.4%)	Yes 43 (100%)	Yes 0	Yes 9 (16%)	Yes 10 (90%)	.013
	No 2 (4.8%)	No 0	No 0	No 1 (4%)	No 1 (1.7%)	No NA	
	Missing 27 (70.7%)	Missing 1 (5.6%)	Missing 0	Missing 22 (96%)	Missing 46 (82%)	Missing 1 (9.1%)	
**CSF hemoglobin (S.E.M.)**	481.0 (106.6)	648.3 (261.2)	465.1 (106.5)	555.6 (183.6)	386.5 (93.1)	370.5 (192.7)	0.85
***n***	36	11	33	23	49	11	
**Postmortem interval (hours) (S.E.M)**95% CI	23.2 (3.15)^b^ 16.82–29.56	46.6 (6.34)^a,d,e^ 33.2–59.99	33.88(3.02)^e^ 27.77–39.98	21.40 (3.85)^b^ 13.43–29.37	20.70 (2.31)^b,c^ 16.1–25.32	24.99 (4.05) 15.98–34.0	<.001
***N***	40	18	42	23	56	11	
**PMI range (Max-Min)**	80.5.00–1.75	101.00–5.5	96.00–2.00	96.00–5.83	99.15–1.75	48.00–3.00	
**RIN (S.E.M.)** ***n***	6.2 (0.2) 35	5.76 (0.5) 16	5.6 (0.2) 31	5.7 (0.5) 12	5.8 (0.2) 39	4.7 (0.4) 10	0.15
**pH (S.E.M.)** ***n***	6.14 (0.08) 26	6.14 (0.10) 14	6.17 (0.1) 24	6.07 (0.13) 11	6.08 (0.06) 36	5.97 (0.8) 6	0.83

Data are presented mean with standard error of the (S.E.M.), years for age at death, and contact sports exposure and as #yes/#no (%) unless otherwise indicated

CERAD plaque density was rated as none (0), sparse (1), moderate (2), or frequent (3) for neuritic plaques

*Int/High* intermediate/high, *AD* Alzheimer disease, *CERAD* Consortium to Establish a Registry for Alzheimer’s disease, *CTE* chronic traumatic encephalopathy, *FTLD* frontotemporal lobar degeneration, *LBD* Lewy body disease, *RIN* RNA integrity number

^a^Different from no CTE/no AD (*p* <.05, Bonferroni corrected)

^b^Different from low CTE (*p* <.05, Bonferroni corrected)

^c^Different from high CTE (*p* <.05, Bonferroni corrected)

^d^Different from low AD (*p* <.05, Bonferroni corrected)

^e^Different from intermediate/high AD (*p* <.05, Bonferroni corrected)

^f^Different from CTE+AD (*p* <.05, Bonferroni corrected)

^#^ANOVA with Bonferroni correction

*χ^2^ test for proportions between all pathology groups

Participants were divided into pathological groups based on NIA-Reagan Criteria and CTE stage as follows. Participants with no evidence of CTE and no elements of NIA-Reagan were labeled as “No CTE/no AD” and were used and referenced as the control group throughout. Participants with CTE Stage of I–II were determined to have early stage disease and termed “Low CTE,” while those with CTE Stage of III–IV were determined to have late stage disease and were termed “High CTE.” Those with no evidence of CTE and NIA-Reagan of high or intermediate probability were termed “Intermediate/High AD,” while those with no evidence of CTE and NIA-Reagan of low probability were classified as “Low AD.” Subjects with CTE and intermediate or high probability of AD were combined to “CTE+AD.”

### CSF sampling and analysis

CSF was obtained post-mortem from the foramen magnum by gently lifting the frontal lobes to access with a large bore needle. CSF was then mixed by gently inverting the tube 5 times. The tubes were centrifuged at 1500 g for 15 min at 4°C. The CSF supernatant was removed with a transfer pipet and aliquoted into 1.5mL microcentrifuge polypropylene tubes. CSF was stored at −80 C prior to use. CSF was then diluted 1:2 with 1% Blocker A (MSD, Rockville, MD, USA, #R93BA) in wash buffer. Immunoassay was performed for Aβ_1-42_ and Aβ_1-40_, using a multiplex plate from MSD (#K15200E), as well as for levels of p-tau_231_ and total tau (MSD #K15121D) according to manufacturer’s protocol. To capture tau phosphorylated at Thr residue 181, antibody AT270 was used and the detecting antibody was the biotinylated HT7 that recognizes residue 159–163 of tau (Thermo Scientific, Rockford, IL). For hemoglobin quantification, CSF was diluted 1:3000 and applied to the RayBio Human Hemoglobin ELISA kit (# ELH-Hgb). All standards and samples were run in duplicate.

### Tissue sampling and analysis

The buffer conditions, protease inhibitors, and centrifugation protocols have been reported previously [11]. A 4-mm-tissue punch was used to isolate and remove gray matter from the gyral crests and sulcal depths of the middle frontal gyrus and neighboring sulci and superior temporal gyrus and sulcus. The brain tissue was homogenized in five-fold volume of 5 M Guanidine Hydrocholride/50 mM Tris-HCL, pH 8.0, with protease inhibitors (Thermo Scientific, 78439) and phosphatase inhibitors (Sigma, P5726 and P0044). The tissue was homogenized using a mechanical homogenizer for 25 strokes followed by ultrasonic disruption on ice. The homogenates were shaken at room temperature overnight. The lysate was diluted 1:80 with 1% Blocker A (MSD, #R93BA) in wash buffer, and immunoassay was performed for Aβ_1-42_ using a multiplex plate from MSD.

### Statistical analysis

Statistical analysis was performed using SPSS 26.0 (IBM Corp, Armonk, NY) and Prism v8 (Graph-Pad Software, La Jolla, CA). A one-way analysis of variance (ANOVA) was used to compare age among groups. Levels of Aβ_1-40_, Aβ_1-42_, p-tau_181_, p-tau_231_, and total tau that were outside 3X the interquartile range were eliminated as outliers and included *n*=1 from no CTE/no AD (control) group, *n*=1 from low CTE group, *n*=1 high CTE group, *n*=1 from low AD group, and *n*=1 from intermediate/high AD group for ptau 181 analysis, *n*=3 from the control group, *n*=1 from low AD group, *n*= 1 for the high CTE group, *n*=6 from intermediate/high AD group, and *n*=1 from CTE+AD group for ptau 231 analysis, *n*=1 from no CTE/no AD (control) group, *n*=2 from high CTE group for total tau analysis, *n*=5 from control group, *n*=3 from low CTE group, *n*=4 from high CTE group, *n*=1 from intermediate/high AD group, *n*= 1 for the CTE+AD group for Aβ_1-42_ analysis, *n*=2 from the control group, *n*=1 from low CTE group, *n*=3 from high CTE group, *n*=1 from low AD group, and *n*=2 from intermediate/high AD group for Aβ_1-40_ analysis. A two-sample chi-square test weighted by sample size was used to compare the frequency of men in each pathologic group, as well as the frequency of FTLD and LBD pathologies between pathologic groups. CSF analyte levels were used in a Kruskal-Wallis test performed to compare levels of analytes between pathologic groups. CSF measures were also rank-normalized (supplementary Figures e-1 and e-2) and used in one-way ANCOVAs in order to correct for age as a covariate to compare relative amounts of biomarkers between groups (supplementary results).

Statistical significance was set to *p*<0.05 following adjustments for multiple comparisons for all planned analyses. Binary logistic regression analyses were used to determine association between p-tau_231_ and Aβ_1-42_ and CTE or AD pathologic diagnosis controlling for age, sex, PMI, and other variables where appropriate. Linear regressions were performed to determine the relationship between CSF and brain Aβ_1-42_ levels in AD and CTE. Receiver operating characteristic (ROC) curve analysis was used to determine sensitivity and specificity of CSF analytes between diagnoses.

## Results

### Study population

Participants were grouped based on the presence or absence of CTE and/or AD pathology. Group demographic differences for age at death, post-mortem interval (PMI), sex, RNA integrity number (RIN), pH, and presence/absence of FTLD and LBD pathologies are listed in Table 1. Pathologic groups differed in age at death (*p* <0.001) and PMI (*p* <0.001) (Table 1). The low CTE group (*M*= 64.6± 4.6) was younger than the low AD (*M*= 86.7±1.1, *p* <.05), intermediate/high AD (*M*= 81.1±1.3, *p* < 0.05), and CTE+AD groups (*M*= 79.6 ± 2.8, *p* <0.05). The low AD group was older than the high CTE and control groups. The low CTE group had significantly longer PMIs (*M*= 46.6±6.4) than the control (*M*= 23.2±3.15 *p*<0.001), low AD (*M*= 21.4±3.85, *p* <0.001), and intermediate/high AD group (*M*= 20.7±2.3, *p* < 0.05). The high CTE group (*M*= 33.88±3.02) had significantly longer PMIs than the intermediate/high AD group (*M*= 20.7±2.3, *p* < 0.05). Despite these differences in PMI, there was no difference in RIN, pH, or CSF hemoglobin between groups. A majority of men were present in each pathological group for all groups except for the intermediate/high AD group which had a majority of women. Years of contact sports differed between groups (*p* =0.013) for the subset of participants that had this history taken (*n*=95); post hoc pairwise comparisons revealed the control group (*M*= 9.46±2.97) had decreased years of contact sports compared to mild CTE group (*M*= 20.85±3.43, *p*=0.036 Bonferroni-corrected).

#### All groups

Comparing all pathological groups using the Kruskal-Wallis test, there were significant differences in p-tau_231_ H(5)= 16.28, *p*=0.006 and Aβ_1-42_ H(5) = 19.32, *p*=0.002. The low CTE group had significantly higher amount of p-tau_231_ (mean rank= 121.1) compared to the Int/high AD group (mean rank= 74.2, *p*=0.014). Both the high CTE group (mean rank= 58.74) and low CTE (mean rank =58.14) groups were found to have significantly lower levels of Aβ_1-42_ compared to no CTE/no AD (control) (mean rank= 102.1, *p*<0.002 and *p* < 0.05, respectively). There was no significant difference in relative amounts of p-tau_181_, total tau, and Aβ_1-40_ between all groups.

#### Early stage disease

**Fig. 1:**
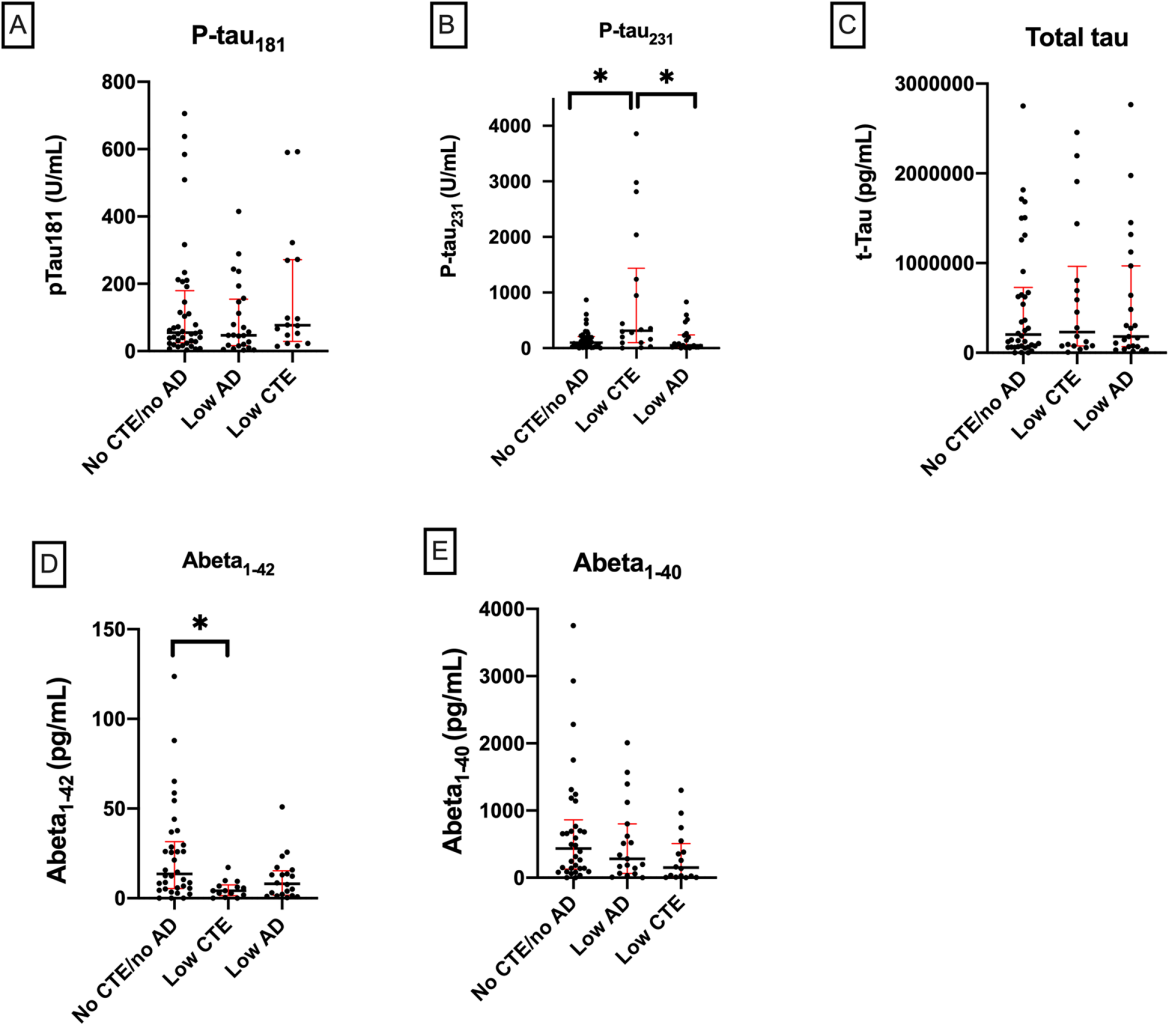
**A** p-tau_181_, **B** p-tau_231_, **C** total tau, **D** Aβ_1-42_, and **E** Aβ_1-40_ for no CTE/no AD (control), low CTE, and low AD groups. Scatter plots show individual values, median, and interquartile range (25–75%) as bars, *p< 0.05 corrected for multiple comparisons; Kruskal-Wallis test

Comparing no CTE/no AD (control), low CTE, and low AD pathological groups using the Kruskal-Wallis test, a difference in p-tau_231_ H(2)= 8.93, *p*=0.015 and Aβ_1-42_ H(2)= 10.27, *p*=0.006 was found. The low CTE group (mean rank= 52.61) had significantly higher levels of p-tau_231_ versus the no CTE/no AD (control) group (mean rank= 35.93, *p*=0.03) and compared to the low AD group (mean rank= 33.02, *p*=0.017) (Fig. 1). The low CTE group (mean rank 22.18) was also found to have significantly lower levels of Aβ_1-42_ compared to the no CTE/no AD (control) (mean rank= 41.51, *p*=0.006) (Fig. 1). The low AD group had lower levels of Aβ_1-42_ compared to the no CTE/no AD (control) group, but this difference was not significant (*p*=0.19). There was no significant difference in relative amount of p-tau_181_, total tau, or Aβ_1-40_ between the no CTE/no AD (control), low CTE, and low AD groups.

Similar results for between group difference for early disease stages were obtained by rank-normalizing CSF measures (supplementary Figure e-1), followed by one-way ANCOVAs in order to correct for age as a covariate (supplementary results).

#### Late stage disease

**Fig. 2:**
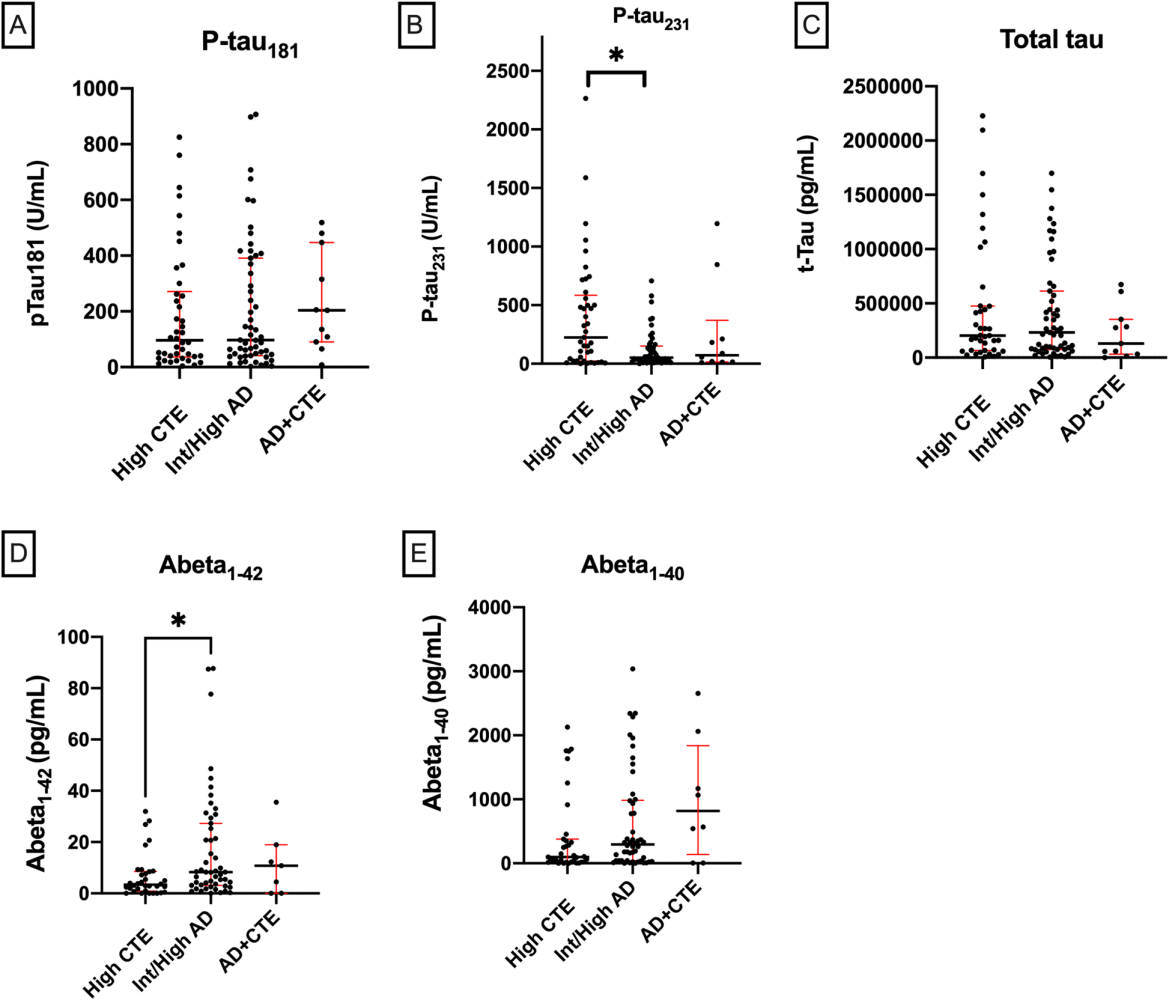
**A** p-tau_181_, **B** p-tau_231_, **C** total tau, **D** Aβ_1-42_, and **E** Aβ_1-40_ for high CTE, intermediate/high AD, and CTE+AD groups. Scatter plots show individual values, median, and interquartile range (25–75%) as bars, *p < 0.05 corrected for multiple comparisons; Kruskal-Wallis test

Comparing high CTE, AD, and CTE+AD pathological groups using the Kruskal-Wallis test, a difference in p-tau_231_ H(2)= 7.096, *p*=0.029 and Aβ_1-42_ H(2) = 7.98, *p* = 0.019 was found overall. The High CTE group had significantly higher levels of p-tau_231_ (mean rank = 59.61) compared to intermediate/high AD (mean rank=43.3, *p*=0.024) (Fig. 2). The High CTE group also had significantly lower levels of Aβ_1-42_ (mean rank= 36.34) compared to the intermediate/high AD group (mean rank =52.99, *p*=0.015) (Fig. 2). There was no significant difference in relative amount of p-tau_181_, total tau, or Aβ_1-40_ between High CTE, AD, and CTE+AD groups. Although there was no significant difference between groups for ptau_181_, all late stage group levels were all numerically greater than the control, indicating that ptau_181_ was elevated in late stage disease above the control group’s levels as expected.

Results for between group difference for late disease stages were also obtained by rank-normalizing CSF measures (supplementary Figure e-2), followed by one-way ANCOVAs in order to correct for age as a covariate (supplementary results).

### Regression analyses

We performed a binary logistic regression analysis to determine the contributions of p-tau_231_ and Aβ_1-42_ in predicting pathological diagnosis of CTE (low and high CTE combined, excluding CTE +AD cases) vs. control. Variables included in the model were p-tau_231_, Aβ_1-42_, age at death, and sex (*n*= 77). Both p-tau_231_ (OR 1.53, 95% CI 1.08–2.16) and Aβ_1-42_ (OR 0.35, 95% CI 0.17–0.74) were significant predictors of CTE status while controlling for age and sex, neither of which were significant predictors (Table 2). Secondary analyses including PMI, pH, and presence of LBD pathology as additional variables did not substantially change the results and were not significant predictors of CTE diagnosis. Although RIN and FTLD were associated with CTE, their presence in the model did not change the associations between ptau_231_, Aβ_1-42_, and CTE status.

**Table 2:** CSF predictors of CTE pathology versus no CTE/no AD (control)

	OR	95% Confidence Interval	*p* value
p-tau_231_ (U/10μL)	1.53	1.08–2.16	0.016
Aβ_1-42_ (pg/100μL)	0.35	0.17–0.74	0.006

Binary logistic regression comparing CTE all stages with no CTE/no AD (control) group while adjusting for age and sex (*n*=77)

We also performed a binary logistic regression to determine the contributions of p-tau_231_ and Aβ_1-42_ in predicting pathological diagnosis of CTE (low and high CTE combined) vs. AD (low AD and intermediate/high AD combined) (Table 3). Variables included in the model were p-tau_231_, Aβ_1-42_, age at death, and sex (*n*= 111). Both p-tau_231_ (OR 1.34, 95% CI 1.02–1.76, *p* = 0.036) and Aβ_1-42_ (OR 0.51, 95% CI 0.28–0.91) were found to distinguish between CTE and AD diagnoses, controlling for age at death (OR 0.91, 95% CI 0.84–0.97) and sex which was not a significant predictor. A secondary analysis adjusting for PMI (*n*= 102), demonstrated that this trend continued but with a decrease in significance most likely due to decreased power, with p-tau_231_ (OR 1.23, 95% CI 0.90–1.72), Aβ_1-42_ (OR 0.60, 95% CI 0.31–1.15), adjusting for age (OR 0.88, 95% CI 0.81–0.97), PMI (OR 1.06, 95% CI 1.01–1.12), and sex, which was not a significant predictor. Additional secondary analyses included the addition of RIN and pH separately as well as presence/absence of FTLD pathology and LBD pathology, none of which were significant predictors of CTE diagnosis.

**Table 3:** CSF predictors of CTE versus AD pathology

	OR	95% confidence interval	*p* value
p-tau_231_ (U/10μL)	1.34	1.02–1.76	0.036
Aβ_1-42_ (pg/100μL)	0.51	0.28–0.91	0.022
Age of death	0.91	0.84–0.97	0.006

Binary logistic regression comparing CTE all stages with AD all stages adjusting for age and sex (*n*=111)

Given the surprisingly low levels of Aβ_1-42_ in CTE, we performed a secondary analysis to test the hypothesis CSF beta-amyloid levels reflect brain tissue Aβ_1-42_ levels in AD, but not in CTE. Using a linear regression it was found that a model including age, sex, and CSF Aβ_1-42_ levels predicted a significant amount of the variance of brain Aβ_1-42_ levels among the AD and control groups combined (F(3, 100)=5.42, *p*=0.002, *R*^2^= 0.14, adjusted *R*^2^= 0.114) and both CSF Aβ_1-42_ (*β* = −0.21, *p* =0.032) and age (*β* = 0.21, *p* =0.036) predicted frontal cortex Aβ_1-42_ while sex (*β* = 0.13, *p* =0.19) did not. In a separate linear regression among the combined CTE and control groups, the overall regression model including CSF Aβ_1-42_ levels, age, and sex predicted a significant amount of the variance of brain Aβ_1-42_ levels (F(3, 77)= 4.52, *p*=0.006, *R*^2^= 0.15, adjusted *R*^2^= .117), though CSF Aβ_1-42_ levels (*β* = −0.17, *p* =0.11) and sex (*β* = −0.14, *p* =0.21) were not found to be a significant predictors of cortical Aβ_1-42_ levels while age was found to be a significant predictor (*β* = 0.38, *p* =0.001).

### Receiver Operating Characteristic curve (ROC) analyses

**Fig. 3:**
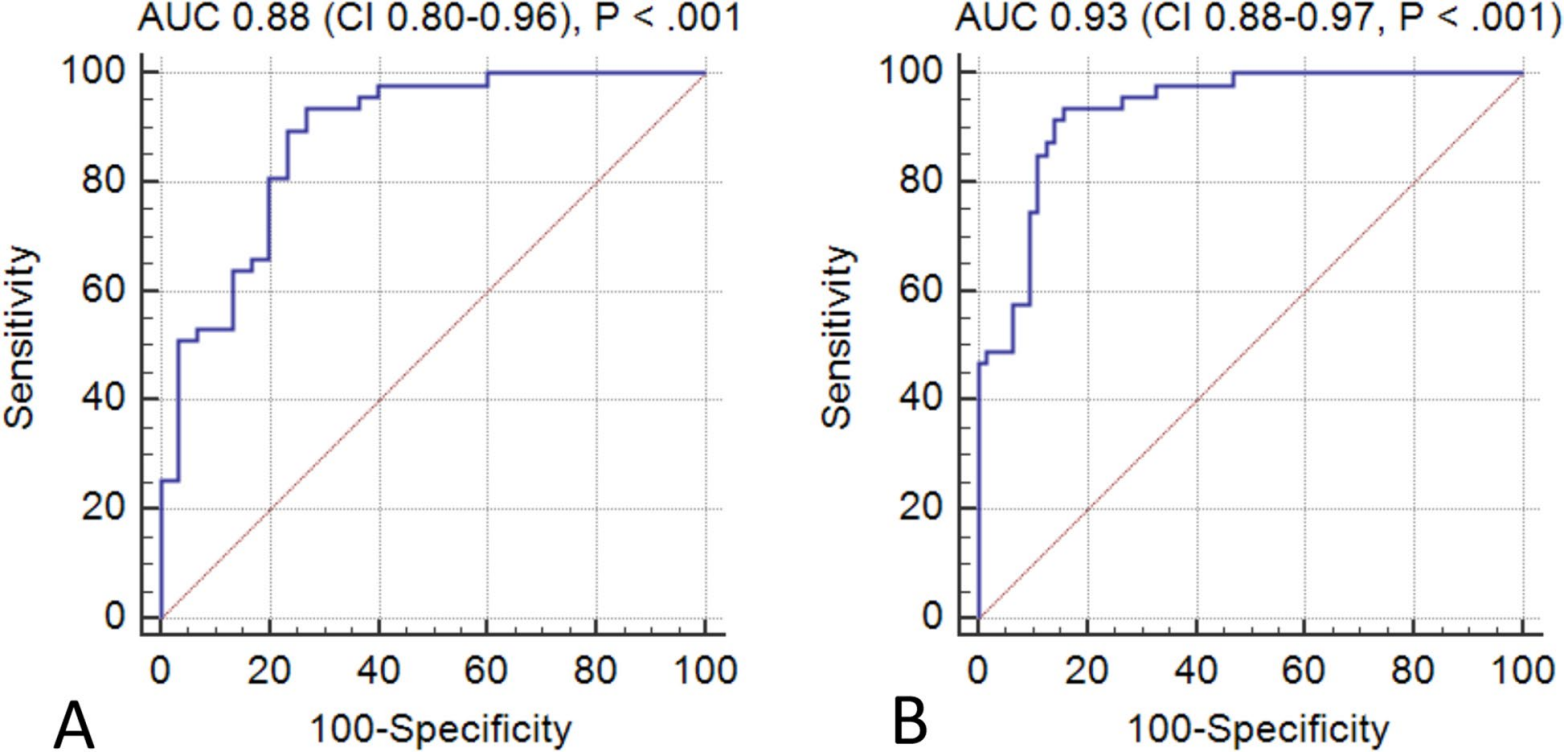
Diagnostic accuracy of **A** p-tau_231_, Aβ_1-42_, age at death, sex for CTE vs. no CTE/no AD (control) diagnosis (*n*= 77) and **B** p-tau_231_, Aβ_1-42_, age at death, and sex were predictive of CTE vs. AD (*n*= 111). AUC area under the receiver operating characteristic curve; CI confidence interval

To assess the diagnostic accuracy of CSF p-tau_231_ and Aβ_1-42_ levels, an ROC analysis was performed to determine if p-tau_231,_ Aβ_1-42,_ age at death, and sex were predictive of CTE vs. control diagnosis. Area under the curve (AUC) was 0.88 (SEM= 0.04, *p* <0.001) (Fig. 3A). A separate ROC analysis was performed for CTE vs. AD diagnosis to determine if p-tau_231_, Aβ_1-42_, age at death, and sex were predictive and demonstrated that AUC was 0.93 (SEM= 0.023, *p* <0.001) (Fig. 3B).

## Discussion

In summary, our results indicate that in post-mortem CSF, p-tau_231_ levels were significantly higher in both the low and high CTE groups compared to the no CTE/no AD (control) group and compared to the AD groups. Furthermore, surprisingly CSF Aβ_1-42_ was found to be decreased in both low stage CTE compared to the no CTE/no AD (control) group as well as in high stage CTE compared to Int/high AD. Overall, increased levels of p-tau_231_ and decreased levels of Aβ_1-42_ in combination were found to be significant predictors of CTE compared to controls and AD.

While three repeat and four repeat tau species are present in both AD and CTE [22, 23], unique folding patterns of tau have been reported to predominate in CTE as compared to AD [24]. Beyond a limited number of studies, little is known regarding the exact tau species present in CTE and how they can be distinguished at the epitope level from the tauopathy of AD, though we recently found that both ptau_202_ and p-tau_231_ are associated with increased years of RHI from American football [25]. Increased p-tau_231_ levels in CTE may be due to tau leakage from cells into the CSF, which may be present even relatively early on in the disease course when clinical symptoms are relatively mild. P-tau_231_ was significantly increased in low CTE, and there was a trend towards an increase for high stage CTE groups compared to AD groups, which could reflect increased neuron and axonal damage in CTE. Studies in AD have revealed that increased phosphorylated tau levels, including both p-tau_181_ and p-tau_231_, correlate with neocortical neurofibrillary burden in AD [26, 27], though a similar relationship in CTE between elevated CSF p-tau_231_ levels and increased p-tau accumulation in the neocortex has not been well-investigated. While t-tau may be a more general marker of neurodegeneration reflecting axonal injury, CSF p-tau_181_ and p-tau_231_ may be specific for the intraneuronal tau pathology seen in both CTE and AD. Our findings from the current study suggest that p-tau_231_ CSF levels may be more sensitive and specific than both t-tau and p-tau_181_ in distinguishing CTE from non-CTE and non-AD participants as well as in distinguishing CTE from AD.

The role of Aβ accumulation in CTE pathophysiology is not well understood. In the current study, the low CTE group exhibited lower Aβ_1-42_ levels in post-mortem CSF compared to those in the control group and surprisingly the High CTE group had decreased Aβ_1-42_ levels compared to the intermediate/high AD group. Decreased CSF Aβ_1-42_ in combination with elevations in tau species and relatively unchanged Aβ_1-40_ are well-established markers of AD and predict the conversion of mild cognitive impairment (MCI) to AD [28–30]. In vivo studies of former NFL players with objective memory deficits have not been found to have increased Aβ deposition compared to controls as measured by florbetapir PET, indicating that cognitive decline in former players at risk of possible CTE was not related to AD or Aβ deposition [31]. In contrast, post-mortem evidence has shown that Aβ accumulation can occur in CTE; however, it is not a consistent pathologic feature and is absent in about half of cases, tending to form diffuse rather than neuritic plaques when present [11]. However, there does appear to be an age-dependent acceleration of Aβ deposition in CTE compared to a normal autopsy population [11]. Furthermore, RHI associated with CTE likely damages blood vessels and is associated with the development of frontal leptomeningeal cerebral amyloid angiopathy (CAA) which is distinct from AD [32]. This preferential localization of Aβ associated with CAA in the leptomeningeal vessels suggests that the mechanism of decreased Aβ_1-42_ seen in CTE may be related to impaired CSF clearance of Aβ, rather than to amyloid sequestration in plaques. We found that there was a significant relationship between CSF and brain levels of Aβ_1-42_ in AD, but not between CSF and brain levels in CTE. This may indicate that sequestration of Aβ_1-42_ in plaques may drive CSF levels of Aβ_1-42_ in AD, but that CSF Aβ_1-42_ is determined by a different mechanism in CTE.

The relationship of the two pathologies underlying AD and CTE is not well understood and as a starting point this study has sought to investigate the levels of several CSF AD biomarkers in the CTE population with the general hypothesis that alterations in these analytes would allow improved discrimination of AD and CTE. Older individuals with CTE pathology are more likely to have concurrent amyloid-beta (Aβ) plaques consistent with AD [16] and may also develop Aβ accumulation at a younger age than individuals without head injuries [11]. In prior studies 13 % of neuropathologically confirmed CTE cases also had AD pathology [33]. In cases where CTE co-occurs with AD, it is unclear if individuals developed a mixed pathology neurodegenerative disorder or whether there is a synergistic relationship between the two pathophysiological processes. An additional possibility is that decreases in CSF Aβ may not correlate as closely with increased Aβ plaque deposition in CTE, a potential departure from the well-established inverse correlation between CSF Aβ levels and Aβ plaque burden in AD. Although we did not find overall group differences in p-tau_181_ and t-tau between any of the pathologic groups, the intermediate/severe AD did show the expected increases in p-tau_181_ and t-tau compared to the no AD/no CTE group.

When controlling for age and sex, both p-tau_231_ and Aβ_1-42_ were able to discriminate CTE from controls using binary logistic regressions. p-tau_231_ and Aβ_1-42_ were still significant when controlling for PMI, RIN, or pH individually. PMI, RIN, and pH all generally correlate with one another. p-tau_231_ and Aβ_1-42_ were also able to discriminate CTE from AD when controlling for age and sex, but lost significance when controlling for PMI, possibly related to decreased power.

### Limitations

This study is limited in that age is a major risk factor for neurodegenerative conditions and the CTE groups were younger than the AD groups. As a result, age at death was included in the regression analysis but there is still the possibility that age could act as a potential confounder in the current study. Differences in years of play generally differ by diagnosis with CTE groups having increased years of play exposure; however, this variable was missing in a significant percentage of cases and as such contact sports play is a potential confounder that could not be accounted for in our models. Furthermore, the groups did not have individuals of each gender evenly distributed and the AD group had increased numbers of women compared to the CTE groups which did not have women. This is a concern as gender differences in neuropathology have previously been reported [34]. However, we have attempted to correct for this in part by running regression and sensitivity analyses among men only and have found similar results for both ptau_231_ and Aβ_1-42_ compared to analyses run with both men and women. In addition, this study was autopsy-based and thus potentially subject to selection bias as individuals whose brains are donated by family may not represent the population more broadly. However, grouping by known pathology allows for definitive associations not possible in a clinical sample where the pathology is unknown. The current study was also limited in that it is post-mortem, and CSF abeta and total tau levels are known to be altered from antemortem levels in post-mortem samples [35]. Both the low and high CTE groups had significantly longer PMIs than the no CTE/no AD (control), low AD, and intermediate/high AD groups which raises concern that a longer PMI could lead to increased tau levels in the CTE groups. We attempted to take this into account by controlling for PMI when possible. Furthermore, given its post-mortem nature, the current study serves not to set a definitive cut-off but to warrant ante-mortem comparison of these amyloid and tau levels in CSF among patients with suspected CTE and AD. Of note, t-tau, p-tau_181_, and Aβ_42_ have been previously evaluated during life in former professional American football players, at increased risk of CTE, and it was found that increased cumulative head impact exposure in former players predicted t-tau levels [36].

Several promising modalities have been investigated for their use as in vivo CTE biomarkers including PET imaging of p-tau [31], peripheral blood levels of total tau and exosomal tau [37, 38], and CSF biomarkers [39, 40]. CSF biomarkers are particularly promising as they closely reflect the dynamic relationship of solute clearance in the glymphatic space [41] and thus may provide a window to relatively early neuropathological changes. Future studies should investigate other tau isoforms that have recently shown promise in AD including p-tau_217_ [42] as well as plasma biomarkers of tau isoforms [7, 43, 44].

## Conclusions

Overall, the current study revealed that levels of post-mortem CSF p-tau_231_ and Aβ_1-42_ were selectively altered in groups with CTE compared to those with AD, and compared to those without CTE and without AD. Both p-tau_231_ and Aβ_1-42_ were also predictive of CTE diagnosis compared to AD group and compared to non-CTE/non-AD groups, indicating that p-tau_231_ represents a potentially sensitive and specific biomarker of CTE, and that decreases in CSF Aβ_1-42_ should be further investigated in vivo among possible CTE patients.

## Supplementary Information

**Additional file 1.** Supplemental results

**Additional file 2: Figure e-1.** Rank-normalized fold change of A. p-tau_181_, B. p-tau_231_, C. total tau, D. Aβ_1-42_ and E. Aβ_1-40_ for no CTE/no AD (control), Low CTE, and Low AD groups. Scatter plots show individual values, median and interquartile range (25-75%) as bars, *p < 0.05 corrected for multiple comparisons; ANCOVA adjusting for age.

**Additional file 3: Figure e-2.** Rank-normalized fold change of A. p-tau_181_, B. p-tau_231_, C. total tau, D. Aβ_1-42_ and E. Aβ_1-40_ for High CTE, Intermediate/High AD, and CTE+AD groups. Scatter plots show individual values, median and interquartile range (25-75%), *p < 0.05 corrected for multiple comparisons; ANCOVA adjusting for age.

**Additional file 4: Table e-1.** Estimated Marginal Means and SEM in parentheses for rank normalized CSF analyte measurements from No CTE/no AD (control) group; showing ANCOVA adjusted for age, *p* <0.05. **Table e-2.** Estimated Marginal Means and SEM in parentheses for rank normalized CSF analyte measurements from No CTE/no AD (control) group; showing ANCOVA adjusted for age, sex, and PMI, *p* <0.05.

## Data Availability

Anonymized data not published within the article will be shared upon reasonable request.

## Abbreviations

CSF: Cerebrospinal fluid
CTE: Chronic traumatic encephalopathy
AD: Alzheimer’s disease
Aβ: Amyloid-beta
t-tau: Total tau
p-tau: Phosphorylated tau
VA-BU-CLF: Veteran’s Affairs-Boston University-Concussion Legacy Foundation Brain Bank
UNITE: Understanding Neurologic Injury and Traumatic Encephalopathy
BU ADRC: Boston University’s Alzheimer’s Disease Research Center
NACC): National Alzheimer’s Disease Coordinating Center
UDS: Uniform Data Set
FHS: Framingham Heart Study
RHI: Repetitive head impact
LBD: Lewy body disease
FTLD: Frontotemporal lobar degeneration
ALS/MND: Amyotrophic Lateral Sclerosis/motor neuron disease
ANOVA: One-way analysis of variance
PMI: Post-mortem interval
RIN: RNA integrity number
ROC: Receiver Operating Characteristic curve
AUC: Area under the curve
MCI: Mild cognitive impairment
CAA: Cerebral amyloid angiopathy
