# Supplementary material for: A comparison between tau and amyloid-β cerebrospinal fluid biomarkers in chronic traumatic encephalopathy and Alzheimer disease
Source: Alzheimers Res Ther. 2022 Feb 9;14:28. doi: 10.1186/s13195-022-00976-y (PMC8830027; doi:10.1186/s13195-022-00976-y)
Supplement: Supplementary file 1 — Additional file 1. Supplemental results [file 13195_2022_976_MOESM1_ESM.docx]

Supplemental Results

We performed ANCOVAs using rank-normalized CSF data in order to correct for age.

ALL GROUPS

Comparing all pathological groups using a one-way ANCOVA correcting for age, there were significant differences in p-tau_231_ F(5,170) = 3.155, p = 0.009 and Aβ_1-42_ F(5,153) = 3.82, p=0.003. The Low CTE group had an increased amount of p-tau_231_ compared to the control group (p = 0.004), the CTE+AD group (p =0.046), the Low AD group (p = 0.005), as well as the Intermediate/High AD group (p <0.001). There was increased p-tau_231_ in High CTE group compared to the Int/High AD group (p =0.013). There was a trend towards increased p-tau_231_ in the High CTE group compared to controls (p =0.094). Although the omnibus ANCOVA for p-tau_181_ F(5,179) = 1.68, p = 0.14 was not significant, pair-wise comparisons revealed that the Low AD group (p =0.045) had decreased p-tau_181_ compared to the CTE+AD group. Finally, the omnibus ANCOVA for Aβ_1-40_ did not reach significant F(5, 155)= 1.60, p = 0.16, however post-hoc tests revealed that there was decreased Aβ_1-40_ in the High CTE group compared to controls (p =0.026). (Supplemental Table e-1).

The Low and High CTE groups were both found to have significantly lower levels of Aβ_1-42_ compared to controls (p =0.006 and p <0.001 respectively). As expected, Aβ_1-42_ was numerically lower in both Low and Intermediate/High AD compared to controls though these differences were significant only for the Low AD group (p =0.046). The High CTE group had significantly lower Aβ_1-42_ compared to the Intermediate/High AD group (p = 0.006). There was no significant difference in relative amounts of p-tau_181_, total tau, and Aβ_1-40_ between all groups (Supplemental Table e-1). We also performed additional ANCOVAs including age, PMI and sex as covariates to better account for group differences and potential confounders and found similar results (See below).

EARLY STAGE DISEASE

Comparing control, Low CTE, and Low AD pathological groups using an ANCOVA correcting for age, differences in p-tau_231_ F(2,73) = 4.84, p= 0.011 and Aβ_1-42_ F(2,64) = 4.68, p=0.013 were found. The Low CTE group had significantly higher levels of p-tau_231_ versus the control group (p = 0.017) and compared to the Low AD group (p=0.021) (Figure e-1). The Low CTE group was also found to have significantly lower levels of Aβ_1-42_ compared to the control group (p=0.026) (Figure e-1). The Low AD group had numerically lower levels of Aβ_1-42_ compared to the no control group, but this difference was not significant when corrected for multiple comparisons (p=0.17). There was no significant difference in relative amount of p-tau_181_, total tau, or Aβ_1-40_ between the control, Low CTE, and Low AD groups.

LATE STAGE DISEASE

Comparing High CTE, Intermediate/High AD, and CTE+AD pathological groups using an ANCOVA correcting for age, there was a significant difference in the level of Aβ_42_ found F(2,88)= 3.54, p = 0.033 with decreased levels in the High CTE group compared to the Int/High AD group (p= 0.028). There was also a trend in p-tau_231_ (p=0.07) found overall, with trend towards relatively increased p-tau_231_ in the High CTE group compared to the Intermediate/High AD group (p= 0.06) (Figure e-2). There were no significant differences in relative amounts of Aβ_1-40,_ ptau_181_ or total tau between High CTE, Intermediate/High AD, and CTE+AD groups (Figure e-2). Although there are no significant differences between groups for ptau_181_, all late stage group levels were all numerically greater than the control, indicating that ptau_181_ was elevated in late stage disease above the control group’s levels as expected (Supplementary table e-1).

We also performed additional analyses to better account for group differences in PMI, age, and sex.

ALL GROUPS

Comparing all pathological groups using a one-way ANCOVA correcting for age, PMI, and sex there was a significant difference in Aβ_1-42_ F(5,137) = 2.46, p = 0.036. The High CTE group had lower levels of Aβ_1-42_ relative to both the control group (p= 0.001) and also had lower levels of Aβ_1-42_ relative to the High AD group (p=0.011). Although the omnibus ANCOVA for p-tau_231_ F(5,151) = 1.72 p = 0.133 was not significant, pair-wise comparisons revealed that p-tau_231_ was significantly increased in the High CTE group compared to the control group (p= 0.043), and compared to the Low AD (p=0.033) and Intermediate/High AD groups (p= 0.011). Although the omnibus ANCOVA for p-tau_181_ F(5,160) = 1.65, p = 0.149 was not significant, pair-wise comparisons revealed that the CTE+AD group (p =0.038) had increased levels compared to the controls. CTE+AD p-tau_181_ levels were increased compared to the Low AD group (p=0.022). After adjusting for age, PMI, and sex there was no significant difference in relative amounts of total tau or Aβ_1-40_ between all groups (Table e-2).

*Binary Logistic Regressions*

To account for the possibility that group differences in PMI and/or age may be acting as potential confounders, we matched groups for these variables by excluding participants outside of the range of comparison groups and performed additional binary logistic regressions predicting pathological diagnosis of CTE vs. controls. Regression analyses using remaining participants matched for PMI where the variables in the model were p-tau_231,_ Aβ_1-42,_ age and sex (n= 51), where p-tau_231_ (OR 1.47, 95% CI 0.98 -2.21) had a trend towards significance and Aβ_1-42_ (OR 0.42, 95% CI 0.18 -0.99) remained a significant predictor of CTE diagnosis, while age and sex were not significant predictors. Regression analyses were performed using participants matched for age (n= 60), where p-tau_231_ (OR 1.50, 95% CI 1.01 -2.22) and Aβ_1-42_ (OR 0.38, 95% CI 0.17-0.86) remained significant predictors of CTE diagnosis, and neither sex nor PMI were found to be significant predictors.

We also matched groups for age and PMI and performed additional binary logistic regressions predicting pathological diagnosis of CTE vs. AD. Regression analyses using remaining participants matched for PMI where the variables in the model were p-tau_231,_ Aβ_1-42,_ age, and sex (n= 74), where p-tau_231_ (OR 1.2, 95% CI 0.90 – 1.6) had a trend towards significance and Aβ_1-42_ (OR 0.49, 95% CI 0.24 -0.98) and age (OR 0.88, 95% CI 0.80-0.97) were significant predictors of CTE diagnosis, while sex was not found to be a significant predictor. Regression analyses using participants matched for age where the variables in the model were p-tau_231,_ Aβ_1-42,_ PMI, and sex (n= 81), where both p-tau_231_ (OR 1.37, 95% CI 0.99 -1.90) and Aβ_1-42_ (OR 0.56, 95% CI 0.30 – 1.07) trended towards significance as predictors of CTE status and PMI was significant (OR 1.05, 95% CI 1.004 – 1.11), while sex was not found to be a significant predictor.

*ROC Analyses*

The above regressions were then submitted to ROC analyses using matched PMI and age groups respectively and curves were overall similar to that generated without age and PMI matching with similar AUC values. ROCs for CTE vs. controls with PMI matching led to an AUC of 0.88 (SEM 0.04, p<0.001); with age matching generating an AUC of 0.91 (SEM 0.04, p <0.001). Similarly, for the CTE vs. AD model, PMI matching led to an AUC of 0.93, (SEM 0.02, p < 0.001) and finally for this model age matching led to an AUC of 0.94 (SEM 0.02, p < 0.001).

Given that there were also group differences in sex with increased numbers of women in the AD groups, and men in the CTE groups, we also performed sensitivity analyses using male participants alone. Importantly, the AUC for the CTE vs. control comparison including p-tau_231,_ Aβ_1-42,_ and age at death among male participants only yielded an AUC of 0.81 (SEM= 0.05, p<0.001, n= 77); the AUC for the CTE vs. AD diagnosis comparison including p-tau_231,_ Aβ_1-42,_ and age at death among male participants only yielded an AUC of 0.87 (SEM= 0.03, p<0.001, n= 111). Overall, these ROC analyses were not statistically different from those run using the entire sample of both men and women (p > 0.05).

Supplemental figure captions:

Figure e-1. Rank-normalized fold change of A. p-tau_181_, B. p-tau_231_, C. total tau, D. Aβ_1-42_ and E. Aβ_1-40_ for no CTE/no AD (control), Low CTE, and Low AD groups. Scatter plots show individual values, median and interquartile range (25-75%) as bars, *p < 0.05 corrected for multiple comparisons; ANCOVA adjusting for age.

Figure e-2. Rank-normalized fold change of A. p-tau_181_, B. p-tau_231_, C. total tau, D. Aβ_1-42_ and E. Aβ_1-40_ for High CTE, Intermediate/High AD, and CTE+AD groups. Scatter plots show individual values, median and interquartile range (25-75%), *p < 0.05 corrected for multiple comparisons; ANCOVA adjusting for age.
