# Supplementary figures and images for: A comparison between tau and amyloid-β cerebrospinal fluid biomarkers in chronic traumatic encephalopathy and Alzheimer disease
Source: Alzheimers Res Ther. 2022 Feb 9;14:28. doi: 10.1186/s13195-022-00976-y (PMC8830027; doi:10.1186/s13195-022-00976-y)

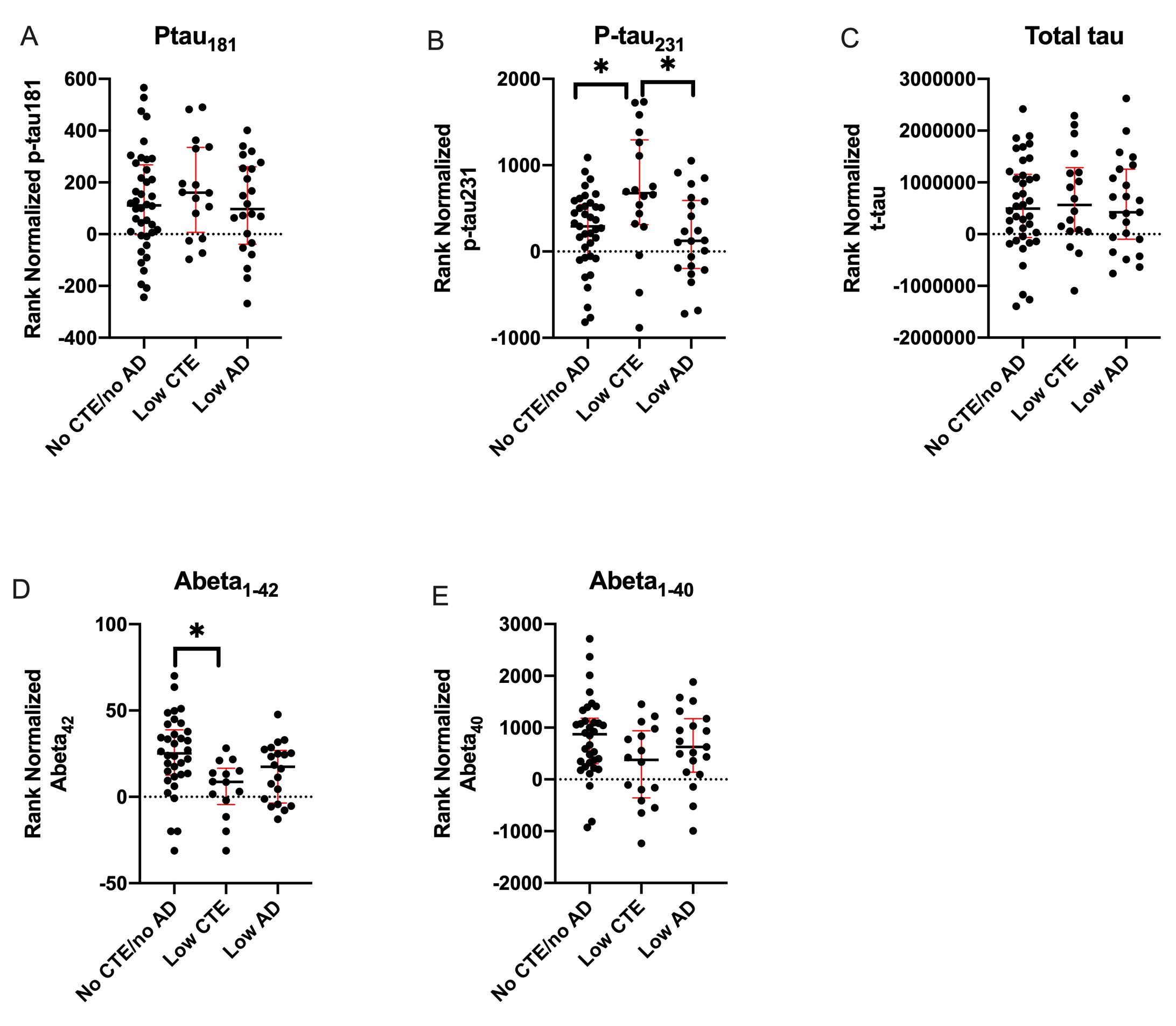

Supplement: Supplementary file 2 — Additional file 2: Figure e-1. Rank-normalized fold change of A. p-tau181, B. p-tau231, C. total tau, D. Aβ1-42 and E. Aβ1-40 for no CTE/no AD (control), Low CTE, and Low AD groups. Scatter plots show individual values, median and interquartile range (25-75%) as bars, *p < 0.05 corrected for multiple comparisons; ANCOVA adjusting for age. [file 13195_2022_976_MOESM2_ESM.tiff]

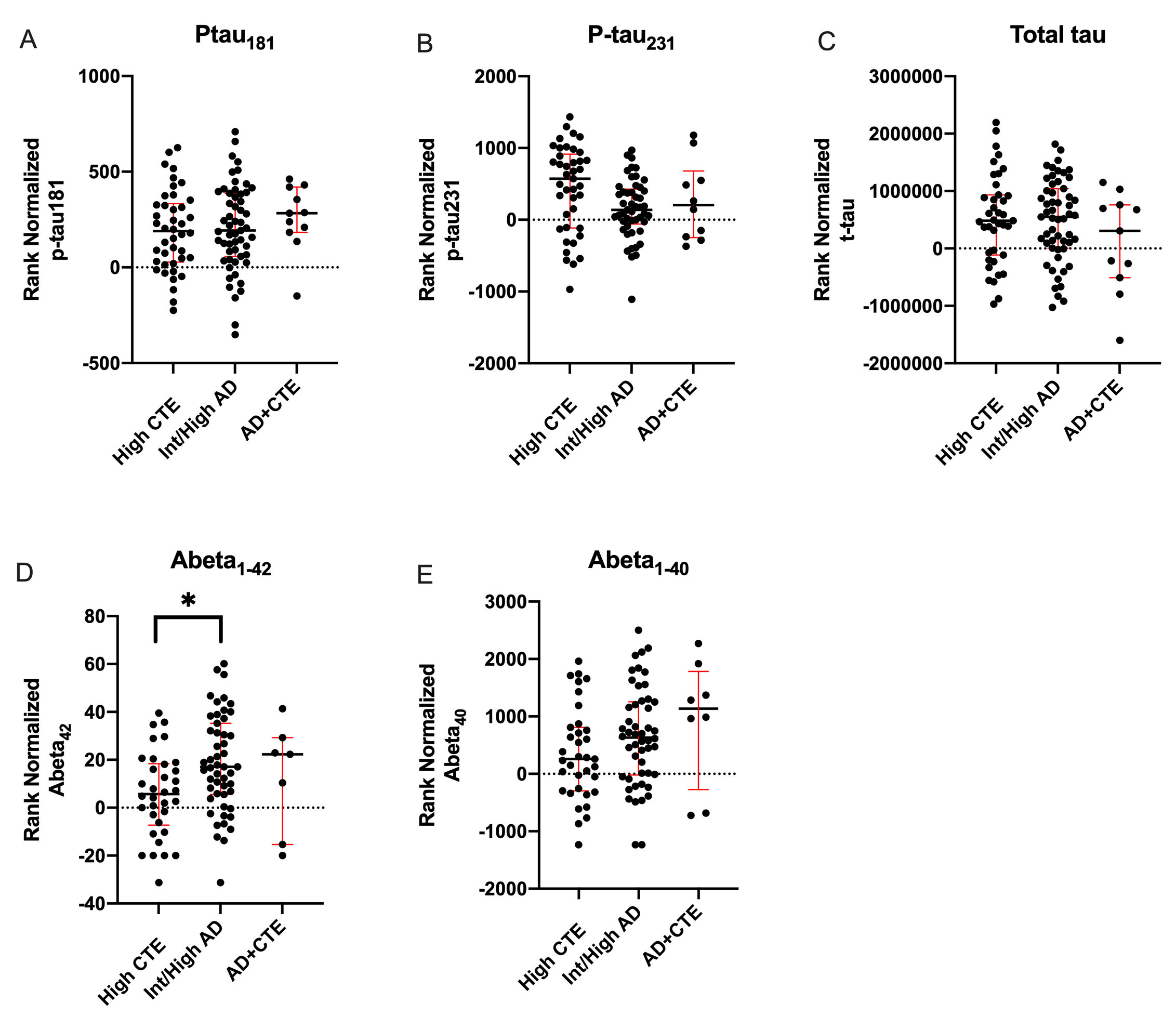

Supplement: Supplementary file 3 — Additional file 3: Figure e-2. Rank-normalized fold change of A. p-tau181, B. p-tau231, C. total tau, D. Aβ1-42 and E. Aβ1-40 for High CTE, Intermediate/High AD, and CTE+AD groups. Scatter plots show individual values, median and interquartile range (25-75%), *p < 0.05 corrected for multiple comparisons; ANCOVA adjusting for age. [file 13195_2022_976_MOESM3_ESM.tiff]
