# Supplementary material for: A comparison between tau and amyloid-β cerebrospinal fluid biomarkers in chronic traumatic encephalopathy and Alzheimer disease
Source: Alzheimers Res Ther. 2022 Feb 9;14:28. doi: 10.1186/s13195-022-00976-y (PMC8830027; doi:10.1186/s13195-022-00976-y)
Supplement: Supplementary file 4 — Additional file 4: Table e-1. Estimated Marginal Means and SEM in parentheses for rank normalized CSF analyte measurements from No CTE/no AD (control) group; showing ANCOVA adjusted for age, p <0.05. Table e-2. Estimated Marginal Means and SEM in parentheses for rank normalized CSF analyte measurements from No CTE/no AD (control) group; showing ANCOVA adjusted for age, sex, and PMI, p <0.05. [file 13195_2022_976_MOESM4_ESM.docx]

|  | No CTE/no AD | Low AD | Low CTE | Int/High AD | High CTE | AD+CTE |
| --- | --- | --- | --- | --- | --- | --- |
| Aβ_1-42_ | 24.69(3.38)^c,b,d^ | 13.14(4.57)^a^ | 6.81(5.57)^a^ | 18.19(2.79)^c^ | 6.0 (3.38)^a,e^ | 13.13(7.43) |
| ptau_231_ | 240.6(86.5)^b^ | 178.0(115.4)^b^ | 691.5(129.9)^a,c,d,e,f^ | 156.0 (76.0)^b,c^ | 440.9(82.3)^d,e^ | 264.5(166.2)^b^ |
| ptau_181_ | 124.9(32.8) | 104.79(45.1)^f^ | 178.4(52.5) | 206.5(27.9) | 192.9(31.9) | 258.9(61.9)^d^ |
| ttau | 545461.7(134365.1) | 562804.8(177267.4) | 683353.7(203026.7) | 562804.8(177267.4) | 515298.8(132139.4) | 111348.5(248211.1) |
| Aβ _1-40_ | 810.7(141.6)^c^ | 602.5(195.4) | 320.4(215.7) | 636.7(117.9) | 363.8(140.1)^a^ | 921.8(291.3) |

Table e-1. Estimated Marginal Means and SEM in parentheses for rank normalized CSF analyte measurements from No CTE/no AD (control) group; showing ANCOVA adjusted for age, *p* <0.05.

^a^Different from No CTE/No AD

^b^Different from Low CTE

^c^Different from High CTE

^d^Different from Low AD

^e^Different from Intermediate/High (Int/High) AD

^f^Different from CTE+AD

|  | No CTE/no AD | Low AD | Low CTE | Intermediate/High AD | High CTE | AD+CTE |
| --- | --- | --- | --- | --- | --- | --- |
| Aβ_1-42_ | 22.8(3.7)^c^ | 12.69(4.58) | 8.85(6.4) | 18.60(3.0)^c^ | 4.87(4.0)^a,e^ | 12.1(7.5) |
| ptau_231_ | 280.2(89.3)^c^ | 220.47(110.0)^c^ | 544.2(136.0) | 205.0 (80.8)^c^ | 543.1(91.8)^a,d,e^ | 271.1(156.9) |
| ptau_181_ | 130.7(34.5)^f^ | 103.2(44.0)^f^ | 165.8(55.4) | 185.7(29.9) | 208.6(36.0) | 275.7(60.4)^a,d^ |
| ttau | 580480.7(140784.2) | 571140.9(170539.5) | 577529.8(211943.8) | 450102.2(118684.7) | 613133.1(148197.6) | 184595.8(239086.3) |
| Aβ _1-40_ | 746.0(151.7) | 571.8(192.8) | 501.6(234.7) | 667.5(127.3) | 470.3(159.5) | 855.9(289.3) |

Table e-2. Estimated Marginal Means and SEM in parentheses for rank normalized CSF analyte measurements from No CTE/no AD (control) group; showing ANCOVA adjusted for age, sex, and PMI, *p* <0.05.

^a^Different from No CTE/No AD

^b^Different from Low CTE

^c^Different from High CTE

^d^Different from Low AD

^e^Different from Intermediate/High (Int/High) AD

^f^Different from CTE+AD
